# Mesenchymal Stem Cells Delivery in Individuals with Different Pathologies: Multimodal Tracking, Safety and Future Applications

**DOI:** 10.3390/ijms23031682

**Published:** 2022-01-31

**Authors:** Carolina Belmar-López, Georges Vassaux, Ana Medel-Martinez, Jerome Burnet, Miguel Quintanilla, Santiago Ramón y Cajal, Javier Hernandez-Losa, Antonio De la Vieja, Pilar Martin-Duque

**Affiliations:** 1 Instituto Aragonés de Ciencias de la Salud/IIS Aragón, 50009 Zaragoza, Spain; carolinabelmar@oncogenomicsperu.com (C.B.-L.); amedel.iacs@aragon.es (A.M.-M.); 2 Institut de Pharmacologie Moléculaire et Cellulaire, INSERM, CNRS, Université Côte d’Azur, 06560 Valbonne, France; vassaux@ipmc.cnrs.fr; 3 Cancer Research UK, Queen Mary University of London, London E1 4NS, UK; jerome.burnet@gmail.com; 4 Instituto de Investigaciones Biomedicas Alberto Sols (CSIC-UAM), 28029 Madrid, Spain; mquintanilla@iib.uam.es; 5 Pathology Department, Hospital Universitari Vall d’Hebron, 08035 Barcelona, Spain; sramon@vhebron.net (S.R.y.C.); jahernan@vhebron.net (J.H.-L.); 6 Endocrine Tumors Unit, Unidad Funcional de Investigación en Enfermedades Endocrinas (UFIEC), Instituto de Salud Carlos III (ISCIII), 28222 Majadahonda, Spain; 7 Centro de Investigación Biomédica en Red de Cáncer (CIBERONC), Instituto de Salud Carlos III (ISCIII), 28029 Madrid, Spain; 8 Fundación ARAID, 50018 Zaragoza, Spain; 9 Networking Research Center in Biomaterials, Bioengineering and Nanomedicine (CIBER-BBN), Instituto de Salud Carlos III, 28029 Madrid, Spain

**Keywords:** mesenchymal stem cells, sodium/iodide symporter (NIS), transdifferentiation, therapy, imaging PET, SPECT, bioluminescence, luciferase, radioiodine therapy, diabetes, injury

## Abstract

Due to their ease of isolation and their properties, mesenchymal stem cells (MSCs) have been widely investigated. MSCs have been proved capable of migration towards areas of inflammation, including tumors. Therefore, they have been suggested as vectors to carry therapies, specifically to neoplasias. As most of the individuals joining clinical trials that use MSCs for cancer and other pathologies are carefully recruited and do not suffer from other diseases, here we decided to study the safety and application of iv-injected MSCs in animals simultaneously induced with different inflammatory pathologies (diabetes, wound healing and tumors). We studied this by in vitro and in vivo approaches using different gene reporters (GFP, hNIS, and f-Luc) and non-invasive techniques (PET, BLI, or fluorescence). Our results found that MSCs reached different organs depending on the previously induced pathology. Moreover, we evaluated the property of MSCs to target tumors as vectors to deliver adenoviruses, including the interaction between tumor microenvironment and MSCs on their arrival. Mechanisms such as transdifferentiation, MSC fusion with cells, or paracrine processes after MSCs homing were studied, increasing the knowledge and safety of this new therapy for cancer.

## 1. Introduction

Despite the development of novel therapeutic methods in recent years, there is a lack of standard and effective therapies for pathologies such as cancer, diabetes, or stroke. This represents a major challenge for public health in all countries. Nowadays, more than 300 million people all over the world are estimated to be affected by neurodegenerative diseases (Huntington’s, Parkinson’s, and Alzheimer’s), diabetes, cardiac diseases, and cancer. Therefore, the scientific community has focused its efforts on developing advanced therapies, such as gene and cell therapies.

Advanced therapies are designed to increase tissue selectivity to avoid the immune response, and to reach high expression levels of the therapeutic gene included in the vector at the area of interest. Nevertheless, one of the key problems of this therapy is the efficient delivery of the therapeutic transgenes to the pathological sites. The vectors commonly used do not carry the mentioned requirements. For that reason, the development of new vectors is essential for the improvement/progression of gene therapy.

Mammalian cells have been proposed as new vector systems based on their characteristics of immune recognition and toxicity. Analysis of the therapeutic efficacy of mesenchymal stem cells (MSCs), including bone marrow (BM) stem cells, has recently been the focus of many kinds of research [1]. Populations of mesenchymal stem cells are present in many adult tissues, acting as cellular reservoirs and aiding renewal of the tissues after trauma, disease, or aging. Bone marrow is the major source of adult hematopoietic stem cells (HSC) that produce circulating blood elements, although it also contains MSCs that are involved in the regeneration of mesenchymal tissues.

MSCs have been studied as vehicles for gene and cellular therapies due to the advantages they have [2,3] of being well-known, easy to extract, expand, and transduce in tissue culture, and extensive and proliferative potential, among others. Small populations of MSCs expressing the corrected gene are able to recover organs, showing them to be a powerful tool in gene therapy for the treatment of numerous disorders. As a result, the number of clinical trials using MSCs to cure damaged areas approaching several pathologies has increased considerably during the last years [4,5].

How MSCs are directed to the damaged area and how they interact with the tissue microenvironment is still unknown. Possibly, at adulthood, these cells act as reparative cells that are mobilized after several damage signals, and are able to migrate to the scar formation area and participate in the regeneration process. A possibility is that chemoattractant signals for MSCs could be immunological signals such as cytokines. Tumor stroma formation can be considered a functionally analogous inflammatory process to wound healing or scar formation based on the ability to secrete cytokines, extracellular matrix molecules, and other essential factors for tumor growth [6,7]. Regarding this, it has been proposed that MSCs are able to migrate to tumor areas because of tumoral stroma cytokine secretion.

Additionally, it has been proposed that MSCs could migrate to other pathologies [8]. These observations and the fact that MSCs are able to transport viruses efficiently let us postulate that MSCs could deliver gene therapy viral vectors to several pathological areas where inflammation processes are ongoing, such as tumors and scars, or produced by diseases such as Parkinson’s, arthritis, or diabetes. Different approaches to regenerative medicine, especially for cancer treatment, have already been reported [9,10]. Nevertheless, the right application of stem cells in the clinic is hampered by our lack of knowledge about how these cells behave in vivo (location and amplitude of gene transfer) on their arrival.

In recent years, functional imaging using positron emission tomography (PET) or single-photon emission tomography (SPECT) have become valuable tools to study human disease in animal models [11]. The limits of these imaging techniques can be compensated with anatomical information obtained from a CT. Whereas PET uses radioactive isotopes emitting positrons (e.g., ^18^F, ^15^O, ^124^I, or ^11^C), SPECT uses isotopes that emit gamma photons, including ^99m^Tc, ^123^I, or ^125^I. The human sodium iodide symporter gene (hNIS) has been broadly used as a reporter gene due to its ability to allow iodide uptake [12,13,14]. The use of hNIS not only offers the advantage of being visualized in techniques such as SPECT/PET by using radioisotopes, e.g., ^124^I, but also provide therapeutic applications with other iodine isotopes such as ^131^I [15]. In the case of ^131^I, high-energy nuclear electron emissions are used to kill hNIS transduced target cells. Ionizing radiation leads to DNA damage, which is primarily caused by both direct and indirect effects of radiation. Direct effects of ^131^I radiation inhibit cell proliferation, enhance cell apoptosis, and promote cell cycle arrest [16]. Additionally, radiation indirectly destroys the surrounding hNIS transduced cells through non-targeted bystander effects [15,17].

In addition, recently in vivo optical imaging has become a widely used tool. The emergence of new technologies such as the IVIS Lumina, designed both for bioluminescent and fluorescent imaging, the improvements in the firefly luciferase (f-Luc), and the onset of multiple spectral variants of the green fluorescent protein (GFP) have completely revolutionized the field [18,19]. Molecular imaging techniques allow cellular tracking, increasing the efficacy and safety of cell therapy treatments in vivo. Nevertheless, little is known about the fate of MSCs once they have arrived in the tumor. In fact, few studies have covered the intrinsic molecular mechanisms supporting MSCs homing. Different hypotheses have been postulated about the fate of these cells, suggesting the possibility of transdifferentiation or cell fusion mechanisms, which may contribute to tumor growth [20,21,22].

Here we propose MSCs as vectors of different cargos to treat cancer, but with the hypothesis that they could also reach other damaged areas.

In recent years, the number of clinical trials using MSCs as vectors for cancer therapies has increased. In those trials selected cancer patients are free from other diseases. Nevertheless, when therapeutic MSCs reach the clinic, the target cancer patients that could benefit from this treatment might be burdened with other diseases. We hypothesize that the presence of other pathologies (especially those involving inflammation) will decrease MSCs therapy efficiency by alteration of MSC migration to tumors. Additionally, in such cases MSCs therapy could be potentially dangerous to those patients. To prove this, we studied MSCs migration to target organs in patients with tumors and inflammation processes produced by diabetes and wound healing. Furthermore, we explored vector safety and efficacy when MSCs reach the tumor, in addition to fusion and/or gen transdifferentiation effects. Interestingly, MSCs biodistribution results were similar regardless of the three reporter genes used, showing that the transgene does not affect homing of the MSCs. In general, our results provide relevant information on the use of MSCs as therapeutic vehicles, both in their migration to damaged areas and in cancer, as well as on their therapeutic effects.

## 2. Results

The first objective was the analysis, in parallel, of MSCs migratory capacity in different inflammatory processes such as diabetes, superficial lesions, and tumor processes. The strategy used to carry it out was through different reporter genes that allowed us to specifically monitor the pathology. Firstly, we used the GFP gene, because its detection is eased by in vitro tissue techniques; secondly, the SLC5A5 gene (that codifies the sodium/iodide symporter (hNIS) protein) because of its theragnostics properties (antitumor therapy by accumulation of therapeutic doses of ^131^I and in vivo visualization by PET-SPECT/CT techniques using ^124^I); and finally, the fLuc gene for visualization in tissues where endogenous hNIS expression (abdominal areas, due to the stomach and urinary bladder’s signals) make it very difficult to track cells by radio-iodide isotopes.

### 2.1. Characterization of the Infectivity of mBMSCs with Different Adenoviral Vectors

As all of our reporter genes were inserted in adenoviral vectors, first, we evaluated the ability of MSCs to be productively infected by those vectors, thus expressing our reporter genes. For this reason, we infected murine bone marrow mesenchymal stem cells (mBMSCs) with adenoviruses expressing sodium iodine symporter (Ad-hNIS), fluorescent GFP (Ad-GFP), or luciferase f-Luc (Ad-fLuc).

**Figure 1:**
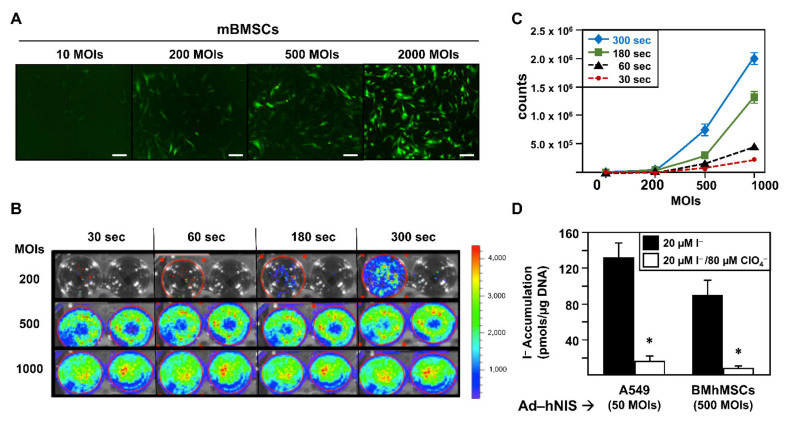
Comparative in vitro infectivity testing of mBMSCs with Ad-GFP, Ad-fLuc, and Ad-hNIS at different MOIs. (**A**) Fluorescence microscopy visualization of mBMSCs infected with Ad-GFP at 10, 200, 500, and 1000 MOIs after 48 h. Images at 10×. Scale bar 100 µm. (**B**) Visualization by IVIS Lumina of mBMSCs infected with Ad-fLuc at 200, 500, and 1000 MOIs. 100 μL of luciferin (final 150 µg/mL) were added 48 h post-infections. Different exposure times were measured (30, 60, 180, and 300 s). Images have a color spectrum, where blue corresponds to the weakest bioluminescence signal and red to the strongest. Exposition times at 30, 60, 180, and 300 s were assayed. (**C**) Representation of the quantification of the bioluminescence signal calculated by delimiting the region of interest or ROI. (**D**) ^125^I uptake of mBMSCs and A549 cells infected with Ad-hNIS at 500 and 50 MOIs respectively after 72 h of infection both in triplicate. The black bars indicate the accumulation of I^−^ (pmols/μg DNA) versus the white bars where hNIS dependent I^−^ accumulation is specifically inhibited by ClO_4_^−^. The statistical analysis was performed with the Kolmogorov–Smirnov test, followed by the Tukey HSD test. (* *p* < 0.05).

We performed an assay using Ad-GFP at different multiplicities of infection (MOIs): 10, 100, 500, and 2000. Figure 1A shows fluorescence images obtained 48 h after infection. As shown, a gradual increase in the GFP signal was detected from 200 MOIs. We next determined Ad-fLuc infection effectivity. Figure 1B reveals results obtained 48 h after Ad-fLuc infection at 200, 500, and 1000 MOIs. We found that luciferase expression could be detected at 500 and 1000 MOIs after 30 s of exposure. At 200 MOIs, luciferase expression could be detected after 300 s of exposure. In fact, the graphic representation of the bioluminescence quantification signal obtained by delimiting the region of interest or ROI (Figure 1C) only exhibited significant differences in the exposure times at 500 and 1000 MOIs. Finally, we examined Ad-hNIS infectivity potential through a ^125^I uptake test using A549 cells as infection control (Figure 1D). While A549 cells were infected at 50 MOIs, mBMSCs were infected at 500 MOIs. In both cases, 72 h later, cells were able to accumulate much more iodide in comparison with inhibited hNIS-mediated iodine flow cells, demonstrating the functional expression of hNIS.

Overall, the results show the ability of mBMSCs to be productively infected by adenoviral vectors at 500 MOIs, a multiplicity of infection four times lower than others described in previous research (2000 MOIs) [23]. In our work, infections with levels of 2000 MOIs showed a very high percentage of cell death 72 h after infection.

### 2.2. Analysis of the Presence of mBMSCs-GFP/mBMSCs-Luc in the Pancreas of Diabetic Mice

After verifying that mBMSCs were capable of being infected by these vectors, we wanted to follow up on the cells, when they were injected in a systemic way. To do this, we conducted a series of in vitro and in vivo follow-up studies using our reporter genes, in animals previously induced with different pathologies, following the Scheme in Table 1.

**Figure 2:**
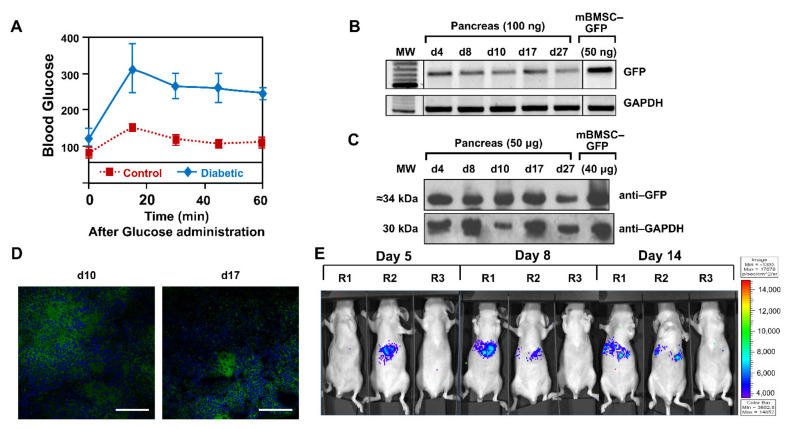
Analysis of the migratory potential of mBMSCs in a diabetic animal model. (**A**) Blood glucose curve of animals that received an intraperitoneal injection of streptozocin. The control animals did not receive the administration of streptozocin, so they were able to normally metabolize the administered glucose. (**B**) Detection of GFP expression in genomic DNA extracted from the pancreas by PCR. Samples of mBMSCs infected with Lv-GFP were used as a positive control for GFP. GAPDH was used as a PCR control. (**C**) Detection of GFP expression by Western blot in protein extracts obtained from the pancreas. Samples of mBMSCs infected with Lv-GFP were used as a positive control of GFP. GAPDH was used as load control. (**D**) Visualization of GFP expression by confocal microscopy in pancreas sections. The images shown correspond to days 10 (40×) and 17 (40×). Scale bar = 50 µm. (**E**) In vivo detection of fLuc expression using IVIS Lumina system. R1 and R2 injected with mBMSCS-fLuc and R3 with PBS. The images have a color spectrum, where blue corresponds to the weakest signal and red to the strongest one.

To confirm the status of the diabetic animals, 10 days after intraperitoneal administration of streptozocin, plasma glucose levels were measured while fasting, and after a glucose overload. Figure 2A shows the glycemia curve of animals that received the administration of streptozocin and control animals, indicating that the administration of streptozotocin caused the necrosis of β cells, therefore a type 1 diabetic process, as previously reported [24].

Once the pathology had been generated, animals (*n* = 18) were separated into three groups: GFP test group (*n* = 10), fLuc test group (*n* = 4), and PBS control group (*n* = 4). They received intravenous injections in the lateral vein of the tail of mBMSCs-GFP, mBMSCs-fLuc and PBS, respectively. In the GFP group, the organs of animal injected with mBMSC-GFP were excised after sacrifice on days 4, 8, 10, 17, and 27. Figure 2B shows pancreas positive expression of GFP, therefore presence of the mBMSCs, by PCR from 4–27 days. Additionally positive expression was observed in lungs up to day 10, but no signal afterwards. Negative expression was observed in liver, heart, and brain (data not shown). Analysis of GFP protein expression by Western blot (Figure 2C) and confocal microscopy (Figure 2D) confirmed those results. In the second test group, mBMSC-fLuc injected animals were analyzed on days 5, 8, and 14 (Figure 2E). Specific signal in the upper abdominal areas (pancreatic area) was observed in test animals (R1, R2) but not in the control animal (R3).

### 2.3. Analysis of the Presence of mBMSCs-GFP/mBMSCs-hNIS in Mice Wounded Skin

A biopsy punch was used to induce a wound, and the animals (*n* = 16) were separated in three groups: GFP test group (*n* = 6), hNIS test group (*n* = 6), and PBS control group (*n* = 4), that received intravenous injections in the lateral vein of the tail of mBMSCs-GFP, mBMSCs-hNIS, and PBS, respectively.

**Figure 3:**
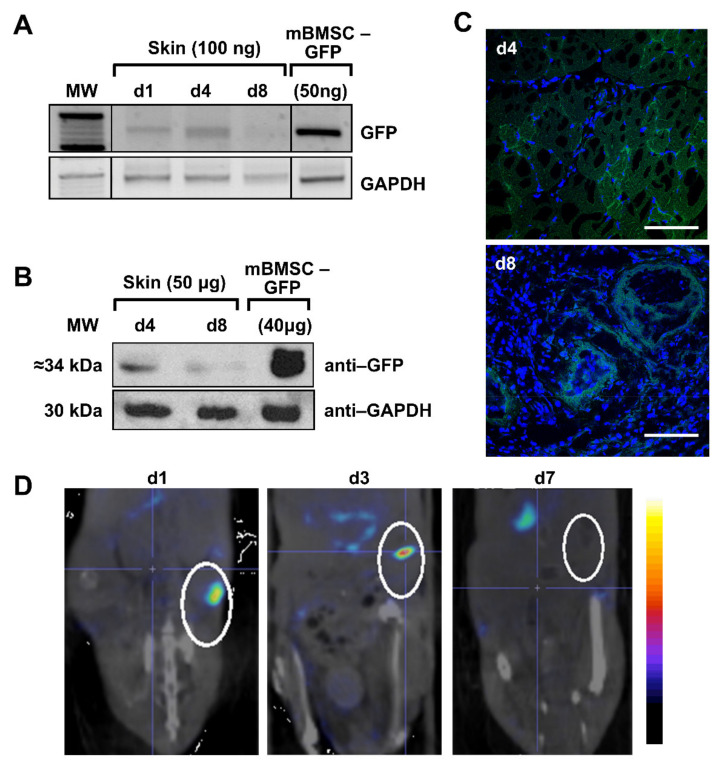
Analysis of the migratory potential of mBMSCs in wound mouse models. (**A**) Detection of GFP expression in genomic DNA extracted from the skin area where the wound was made. Samples of mBMSCs infected with Lv-GFP were used as positive control for GFP. GAPDH was used as a PCR control. (**B**) Detection of GFP expression by Western blot in wounded area protein extracts. Positive control of GFP was used for mBMSCs infected with Lv-GFP. GAPDH was used as load control. (**C**) Confocal microscopy analysis of GFP expression in sections made in the wounded skin. The images shown correspond to days 4 (40×) and 8 (40×). Scale bar = 50 µm. (**D**) In vivo visualization by nanoSPECT of hNIS expression in the wound skin area is located inside the white circles. The wound is located on the right side. Accumulation of ^99m^TcO_4_^−^ (blue corresponds to the weakest signal and red to the strongest) is detected in NIS expressing tissues.

In the GFP test group, positive GFP expression by PCR of wounded skin area was observed in animals sacrificed on days 1 and 4, but low signal was observed on day 8, possibly because the scar was closed (Figure 3A). Lung samples showed positive results throughout the experiment. Liver extracts were positive only on day 4, and the rest of analyzed tissues and timeframes were negative (heart and brain) (data not shown). Analysis of GFP protein expression in skin samples by Western blot (Figure 3B) and confocal microscopy (Figure 3C) confirmed those results.

For imaging, in the hNIS group, animals on days 1, 3, and 7 after injection of the mBMSCs-hNIS were analyzed by nanoSPECT/CT (Figure 3D). In the area of the wound, located in the lower right side of the image, hNIS specific accumulation of ^99m^TcO_4_^−^ can be observed. The most intense signals are found on days 1 and 3. After day 7, the signal is hardly detected, as previously observed by other techniques. As we expected, positive radioisotope signal is observed in the endogenously hNIS expressing tissue (stomach). However, the signal is temporary, since the radioisotope does not accumulate in this tissue, it only passes through [14].

### 2.4. Analysis of the Presence of mBMSCs-GFP/mBMSCs-hNIS in Xenograft Mouse Models

Our last models to study in parallel to previous ones, were neoplasias. When subcutaneous HeLa cell tumors reached the desired size, some animals from this group (*n* = 18) were randomly separated into the three groups: GFP test group (*n* = 10), hNIS test group (*n* = 4), and the PBS control group (*n* = 4). Animals in each test group received intravenous injections of mBMSCs-GFP, mBMSCs-hNIS and PBS, respectively.

**Figure 4:**
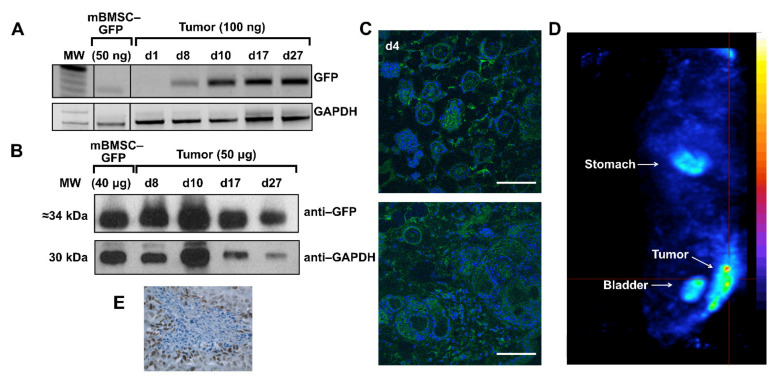
Analysis of the migratory potential of mBMSCs in a xenograft animal model. (**A**) Detection of GFP expression in genomic DNA extracted from tumors. Samples of mBMSCs infected with Lv-GFP were used as a positive control for GFP. GAPDH was used as a PCR control. (**B**) Detection of GFP expression by Western blot in protein extracts obtained from tumors. Samples of mBMSCs infected with Lv-GFP were used as a positive control of GFP expression. GAPDH was used as load control. (**C**) Visualization of GFP by confocal microscopy in tumor sections. The images shown correspond to days 8 (40×) and 17 (40×). Scale bar = 50 µm. (**D**) In vivo detection of hNIS expression in tumors using nanoSPECT. The image shown corresponds to the whole animal in the dorsal position on day 14 after the injection of mBMSCs-hNIS. The tumor is located on the lower right side. Accumulation of ^99m^TcO_4_^−^ (blue corresponds to the weakest signal and red to the strongest) is detected in hNIS expressing tissues (stomach and bladder). (**E**) Expression of NIS by immunohistochemistry in tumor samples.

In the GFP group, organs and tumors of animals injected with intravenous injection mBMSCs-GFP were separated after sacrifice, on days 1, 8, 10, 17, and 27. Figure 4A shows GFP tumors positive expression after day 8. In lungs, GFP expression disappear after the first days and negative expression was observed in liver, heart, and brain (data not shown). Analysis of GFP protein expression by Western blot (Figure 4B) and confocal microscopy in the tumors (Figure 4C) corroborated those results. In the hNIS group, hNIS expression was analyzed by nanoSPECT. Intensive NIS specific accumulation of ^99m^TcO_4_^−^ can be observed in the tumor area (Figure 4D). Expected positive ^99m^TcO_4_^−^ accumulation was observed in both test and control groups in endogenous hNIS expressing tissues such as thyroid, salivary glands, stomach, urinary bladder, and mammary glands [14,25]. As was mentioned before, this signal is temporary, except in the thyroid. Only the tumoral signal was coming from external NIS expression.

In summary, the set of results obtained using these three models shows that mBMSCs are able to migrate in vivo and graft in damaged areas and that, in the case of tumor and diabetes models, they can be detected up to 27 days in the tumor stroma and pancreas, respectively.

### 2.5. In Vivo Therapeutic Effects on Xenograft Tumor Growth after Radioisotope ^131^I Administration

In a previous study, we demonstrated that arrival of MSCs affect proliferation of tumor cells [22]. Additionally, we described several adenoviruses carrying NIS and that the advantage of taking them specifically to the tumors could be differential on a future strategy [22]. In this study we wanted to determine if after using MSCs-hNIS injection, we could use radioactive iodide (RAI) ^131^I accumulated by hNIS to specifically treat the tumor, without affecting other tissues, as it occurs in thyroid RAI [15]. To address this, we performed in vivo RAI assays (^131^I) in animals with xenograft tumors after MSCs-hNIS administration. When subcutaneous HeLa cell tumors reached the desired size, animals from this group (*n* = 16) were separated into four subgroups (each of *n* = 4), as mentioned in Table 2 and injected with their respective treatments. 

**Figure 5:**
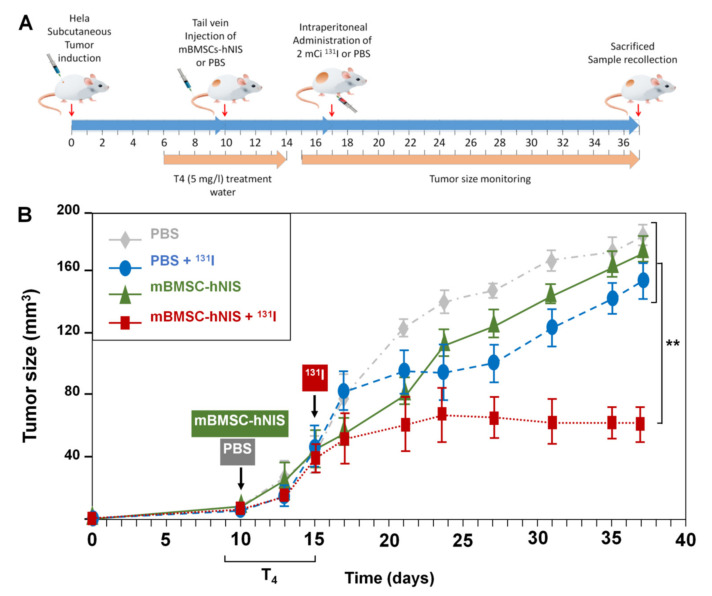
Effect of radio-iodide treatment on xenograft’s tumor growth. (**A**) Time sequence of treatments. Ten days after induction of the HeLa tumors, mBMSCs-hNIS (*n* = 4) or PBS (*n* = 4) was injected intravenously. After the thyroxine (T4) administration period (7 days), 2 mCi of ^131^I or PBS was administered intraperitoneal. During the following 20 days, tumors were measured every 2–3 days. Finally, the animals were sacrificed on day 37. (**B**) Graphic representation of the therapeutic effect of ^131^I treatment. Statistical analysis was performed with the Kolmogorov–Smirnov test, followed by the Tukey HSD test. (** *p* < 0.01).

The time sequence followed during the trial is shown in Figure 5A. Figure 5B shows the growth pattern followed by tumors in the four groups of animals. Until day 15, the tumors in all groups showed a similar growth pattern.

We observed that 20 days after ^131^I or PBS administration, tumor growth continued to a size greater than 150 mm^3^, except in the group treated with mBMSCs-hNIS + ^131^I, where growth and tumor arrest, and even a decrease in tumor size, were observed (Figure 5B and Table 3). These results demonstrated the utility of mBMSCs as vehicles for anti-tumor therapies, especially for adenoviral therapies.

The differences between the different groups were observed from day 21 onwards. Table 3 indicates the average tumor size in animals of the different groups.

### 2.6. Study of the MSCs Fate in the Tumoral Microenvironment

After verifying mBMSCs tropism to tumors and proposing these cells as vehicles of anti-tumor therapy, one of our major interests, was to warranty their safety as vectors. Therefore, we next sought to clarify if, in the tumor, MCSs could undergo processes of cell transdifferentiation or fusion, which may contribute to tumor growth.

First, to explore cell fusion events between MSCs and tumor cells, we used a subcutaneous tumor model of TE671-LoxP/LacZ cells and we intravenously injected BMhMSCs-Cre infected cells, which should migrate towards the tumor. The fusion between both cells would cause the excision of the loxP-STOP-loxP region, thus allowing the expression of LacZ.

**Figure 6:**
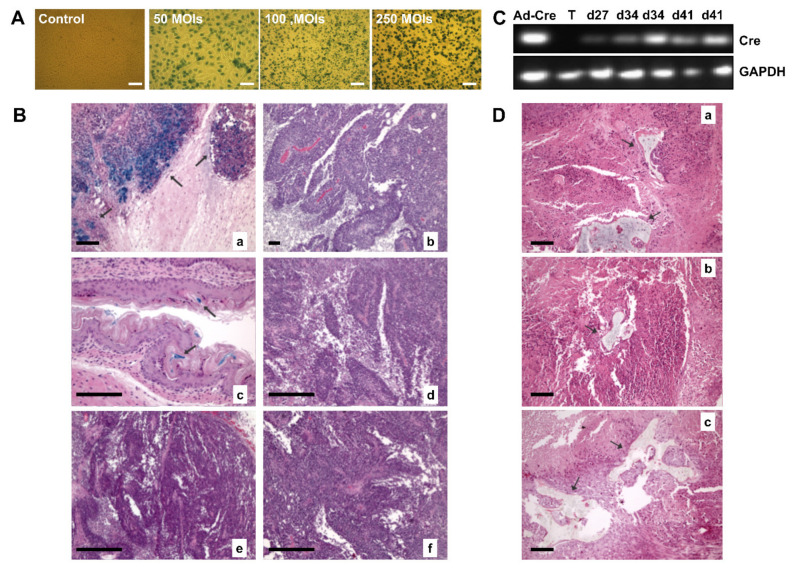
Analysis of hMSCs fate in tumors. (**A**) Detection of LacZ expression in vitro in TE671-LoxP/LacZ cells infected with Ad-Cre. Stained β-galactosidase photographs (10×) were taken 48 h after infection at 50, 100, and 250 MOIs. Scale bar = 100 µm. The control cells were not infected. (**B**) Arrows marked, detection of LacZ expression by β-galactosidase staining in toto in TE671-LoxP/LacZ tumors. Mice that received intratumoral injection of Ad-Cre were used as positive controls. The samples were processed by staining with hematoxylin-eosin and β-galactosidase. The intense blue coloration indicates the expression of LacZ. (**a**) Ad-Cre group images administered intratumorally at day 27 (100×); (**b**) Endogenous expression images of LacZ in the stomach at day 27 (200×); (**c**) Control group images (50×); (**d**) BMhMSCs-Cre test group images at day 27 (50×); (**e**) BMhMSCs-Cre test group images at day 34 (50×); (**f**) BMhMSCs-Cre test group images at day 41 (50×). Scale bar = 10 µm. (**C**) Detection of Cre expression by RT-PCR in TE671-LoxP/LacZ tumors. Tumors that received intratumoral injection of Ad-Cre were used as positive controls. Tumor RNA from the control animals was used as a negative control. GAPDH was used as load control. (**D**) Detection of bone areas in HeLa tumors of animals that received an intravenous injection of hAMCs, hEESCs, and hiPSCs. (**a**) hAMCS-hNIS group images at day 24 (100×); (**b**) hEESCs-hNIS group images at day 24 (100×); (**c**) hiPSCs-hNIS group images at day 24 (100×). Scale bar = 10 µm.

We first evaluated the model, by staining LacZ expression in TE671-LoxP/LacZ cells after the infection with Ad-Cre. Figure 6A shows images of TE671-LoxP/LacZ cells infected in vitro with Ad-Cre at 50, 100, and 250 MOIs. The blue-green color detected with the stain confirms the expression of LacZ in TE671-LoxP/LacZ cells after adenoviral infection.

Then, in vivo studies were performed, separating TE671-LoxP/LacZ xenograft animals (*n* = 13) to be injected in three different conditions: BMhMSCs-Cre test group (*n* = 9), Ad-Cre group (*n* = 2), and control group (*n* = 2), as indicated in Table 4.

Mice were sacrificed after 1 week (day 27), 2 weeks (day 34), and 3 weeks (day 41) after injection. Tumors and other organs were removed. A small sample of each organ was used to extract RNA, and the rest was processed for β-galactosidase staining. To optimize the staining of β-galactosidase, a sample of the stomach was used due to the endogenous expression of LacZ.

As shown in Figure 6B, the samples derived from the group of animals that received intratumoral injection of (10^8^ pFU) Ad-Cre, displayed an intense blue coloration, which seemed to be related to the expression of LacZ in TE671-LoxP/LacZ cells. By contrast, none of the tumors of the animals with intravenous administration of BMhMSCs-Cre showed expression of LacZ.

To corroborate these results and verify that the absence of cell fusion was not related to a lack of distribution and arrival of BMhMSCs-Cre towards the tumor, Cre expression was analyzed by RT-PCR. RNA was extracted from the tumors after animal sacrifice. As reflected in Figure 6C, Cre expression was detected in the group of animals that had received the intratumoral injection of Ad-Cre. This was supported by the results obtained with β-galactosidase staining. However, unlike previous results, signal was detected in the tumor samples of animals from the BMhMSCs-Cre group, with 33% expression in the animals sacrificed during the first week and 66% in those after the second and third weeks. These differences may probably be caused by the distribution of BMhMSCs-Cre in the tumor or the episodic expression of Cre.

Secondly, to verify if there were processes of transdifferentiation after hMSCs arrival, mice coming from the Hela tumor model (*n* = 16) were injected with hMSCs of different sources expressing hNIS (hAMSCS group, HEESCs group, hiPSCs group and a control group, each one with *n* = 4). The use of more types of stem cells, some of them with higher degrees of plasticity (such as hiPSCs), let us know about differentiation mechanisms.

These mice were sacrificed after the acquisition of day 24 using staining with hematoxylin-eosin. Surprisingly, we detected bone formations (Figure 6D) in the tumors of hAMSCs, hEESCs, and hiPSCs groups, demonstrating that hMSCs presence can promote differentiation in a proper tumor microenvironment, regardless of the hMSCs cell tissue.

In summary, these data suggest that, after BMhMSCs-Cre homing in the tumor area, no fusion event occurs between tumor cells and BMhMSCs. Nevertheless, these results argue for a relevant role of transdifferentiation on MSCs tumor asserting.

## 3. Discussion

Due to their properties to migrate to damaged tissues, stem cells have been used as labeled vehicles for therapeutic genes in a widespread range of pathologies such as myocardial infarction or cancer [26,27]. One of the limitations of the applications of stem cell therapies for the global population, after the clinical trials, is the fear of the uncontrolled dissemination of cells in the wrong place and undesired cell growth. Most subjects suffer from more than one pathology at a time, especially at elder ages. Therefore, further studies on the target organs of mesenchymal stem cells for several pathologies would be advantageous.

The detection of gene expression in vivo, in the live subject, could provide information on the location and amplitude of gene transfer, as most gene therapy clinical trials obtain great amounts of information from the current end-points. The use of engineered labeled MSCs to deliver and track genes simultaneously has been promoted as an attractive option. Ideally, this detection should be minimally invasive and should be repeated at certain times in the same patient.

Our work has explored the ability of infected MSCs to migrate in three different pathologies in parallel, with the same source of cells and conditions. We found that labeled MSCs can migrate to and survive in tumor xenografts, pancreas (for diabetes), and skin (when an injury was done) by different approaches. Whereas there was MSCs detection from day 4 in diabetic animals, there was no detection until day 8 in the HeLa tumor model. In both models, there was lung signal until day 10. Curiously, skin injured animals only displayed signal until day 4, when the scar was closed and healed.

The differences between the animal models indicate that there is a MSCs arrival delay depending on the pathology. Other authors have analyzed MSCs migration to wound areas, pancreas, and tumors but in independent studies. When it comes to diabetes, some studies have also detected pancreatic signal 14 days after MSCs double-labeled cells (DAPI-SPIO) transplantation, by magnetic resonance imaging (MRI) [28]. In contrast to our observations, Pan et al. showed that six weeks after transplantation, DAPI fluorescence signal existed in frozen pancreatic sections. Probably, if we had sacrificed animals a few weeks later, these differences would not have been notorious. In all cases, our paper also indicates that BMSCs are mainly distributed in damaged pancreatic tissue in diabetic mouse models.

With regard to skin injured mice, other authors have assessed trafficking and homing of MSCs in response to severe burn wounds. For instance, Liu et al. showed that GFP-labeled human umbilical cord MSCs (hUC-MSCs) transplanted into injured rats migrated into burns from 1 to 3 weeks after transplantation [29]. This group also performed PCR analyses detecting products of human-specific DNA in the wounds of a severe burn group transplanted with hUC-MSCs at weeks 1, 2, and 3. The differences between our results and this study may be influenced by the different type of MSCs employed. In this sense, Kidd et al. studied the migration capacity of mBMSCs in different models of skin wound. In all cases, after intravenous administration, cells were visualized by bioluminescence imaging in target areas from day 3 to day 5 [30]. Along the same lines, Oh et al. reported how mBMMSC/fLuc BLI displayed signals in burn injury lesions 4 days after cell injection, decreasing gradually after this day [31]. This corresponds to our results, since 8 days after mBMSCs administration, no signal was detected in the wound area.

Finally, previous studies have researched MSCs’ migration to tumors. Kidd et al., studied mBMSCs tropism to lung metastasis of MDA-MB-231 cells [30]. Consistent with our study, they were able to detect mBMSCs in lungs from day 3 after intravenous administration until the end of the experiment on day 29, probably due to the method of infusion as all cells infused by the tail vein must pass through the lungs where they can become entrapped. They also analyzed lungs, liver, spleen, and other organs after extracting them from sacrificed animals. These results were also supported by Meleshina et al., who performed a long-term in vivo bioluminescence imaging of intravenously injected BMhMSCs genetically labeled with luc2 in a lung metastases mouse model [32]. As in our results, MSCs showed distribution to lung metastases within the first 2 to 3 weeks. However, the delay in mouse sacrifice allowed them to visualize remigration to the lungs in 6 to 7 weeks. Furthermore, in a similar assay, BMhMSCs expressing fLuc and GFP were confirmed to migrate towards breast and anaplastic thyroid xenograft models in vivo and ex vivo by optical imaging [33]. However, this study only imaged mice 1 h and 24 h after injection. By contrast, Cao et al., who used ferritin gene-based magnetic resonance imaging (MRI) to track tumor tropism of rat MSCs in a rat orthotopic xenograft model of malignant glioma, could only visualize MSCs in vivo for up to 10 days, as confirmed by histology [34]. Notably, recent work has also demonstrated the targeting and distribution of the magnetically labeled rat BMMSCs in rabbit hepatic VX2 tumors, visualizing a signal only 7 days after injection [35]. These inconsistent results in MSCs tumor signal may be related to the labeling of the cells that are often degraded, diluted, and excreted as cell populations divide.

In summary, these data suggest that receptors or factors expressed and secreted by injured tissues facilitate the migration, adhesion, and infiltration of MSCs to the inflammatory site independently of the pathology [36]. MSCs tropism to various pathologies supports the value of using exogenous MSCs as biological carriers for cancerous diseases. However, it should be taken into account that this application in individuals with other inflammatory diseases (e.g., arthritis) could end up with MSCs in a different target tissue than the initially planned. However, as exposed in previous studies, MSCs may show bidirectional and divergent effects on tumor growth when administered systemically. Therefore, in this study, we aimed to offset these effects by loading MSCs with anti-tumor therapies.

In this sense, MSCs have been previously modified to allow the expression of antiangiogenic proteins [37,38] or interferon β in different tumor models such as gliomas, breast cancer, melanoma, or colorectal cancer [39,40]. The use of these MSCs-IFN suppressed the growth of tumor cells by inducing cells to suffer a stack in phase S, increasing apoptosis, thus reducing the size of tumors and increasing the survival rate. MSCs have also been modified by using viral vectors to express several interleukines [41,42], the apoptotic TRAIL transmembrane protein [43], and suicidal genes such as herpes simplex herpes thymidine kinase (HSV-TK) combined with ganciclovir [44]; or by loading them with drug nanoparticles such as paclitaxel and curcumin nanoparticles [45].

Our study was also focused on the engineering of MSCs with suicide gene therapy but using hNIS. Interestingly, we observed very promising results of tumor reduction following treatment with ^131^I in our model. Our results were even better than those obtained by Dywer et al., who used ^131^I therapy in BMhMSCs previously infected with an AdhNIS in a breast cancer model generated with MDA-MB-231 cell [46]. In addition, Knoop et al. explored the application of hNIS as a theranostic gene [26,47,48]. They found that ^124^I accumulation was confined to the tumor and its metastasis by ex-vivo biodistribution analysis of NIS-eMSCs in hepatocellular xenograft model and liver metastasis. Additionally, they observed that tumor growth was 1/3 of controls, and more importantly, the reduction was associated with a significantly extended life span. The differences between those studies and ours may be due to the tumor model used, the source of the stem cells, the initial size of the tumor, and the time of ^131^I application. Together, these results confirm the usefulness of hNIS gene as a visualization tool for monitoring cells using SPECT/PET and radioisotopes such as ^99m^Tc or ^124^I and its use as a therapeutic tool by treatment with ^131^I.

This therapy combined with adenovirus carrying NIS could be a powerful tool for cancer theragnostics as both therapy and diagnostics could be done at the same time, helped by the viral replication in the tumoral area. Nevertheless, it is important to understand the fate of injected cells when they reach the tumors for the safety of the therapy. As far as we know, only a few studies have covered MSCs mechanisms when recruited in tumors. Once there, they are able to interact with tumor cells and with resident non-tumor cells of the stroma, such as immune system cells and endothelial cells supporting vasculogenesis, while secreting cytokines and growth factors. Several hypotheses have suggested the possibility of these cells suffering some events of transdifferentiation [49,50], or cell fusion [38,51] in response to this microenvironment factors. Some authors have shown spontaneous hybrid formation between BMhMSCs and breast cancer cells (MDA-MB-231) [52,53]. Surprisingly, hybrids showed typical MSCs and MDA-MB-231 cell characteristics, a mixed gene expression profile, and an increase in the number of ploidies, as well as increased invasive and metastatic capacity. In the same line, other works have suggested that cell fusion between lung cancer cells and MSCs offered enhanced metastatic capacity and characteristics of cancer stem cells by undergoing EMT [54]. By contrast, Wang et al. revealed that cell fusion between hUC-MSCs and carcinoma cells generates hybrids with a lower growth rate, higher levels of apoptosis, and inhibition of tumorigenesis [55,56].

Contrary to these previous studies, our results show that after BMhMSCs arrival, no cell fusion event occurs. However, we cannot claim that there are no other processes of cell fusion between MSCs and other elements of the tumor-stroma, perhaps promoting tumorigenesis and tumor growth. Ferrand et al. demonstrated the ability of MSCs both in vitro and in vivo to acquire epithelial characteristics through mechanisms of cell fusion with gastric and intestinal epithelial tumor cells [57]. In this process, MSCs suffered the loss of expression of their typical markers, suggesting the possibility of this being a process prior to their reprogramming and subsequent differentiation to epithelial cells. We found that tumors receiving intravenous administration of hAMCs, hEESCs, and hiPSCs, showed bone formations, confirming the ability of these cells to differentiate themselves to cells of the same mesenchymal lineage. This is supported by Wang et al., who found that MSCs that migrated to lung tumors differentiated into osteoblasts [58].

In summary, our results demonstrate that MSCs naturally migrate to damaged areas produced by three different pathologies such as diabetes, wound healing, and tumors. Thereafter, these MSCs cells, loaded with different gene reporters (GFP, f-Luc, and hNIS), allow us to track their migration and differentiation with non-invasive techniques (PET, SPECT, BLI, and fluorescence). Remarkably, we also demonstrated that MSCs, combined with hNIS gene, can be a very powerful anti-tumor strategy using ^131^I therapy. This therapy allows not only the direct destruction of tumor cells but also the surrounding malignant cells within the tumor stroma by the bystander effect. Finally, we found cell fusion absence of MhMSCs and tumor cells, but clear transdifferention of BMhMSCs into bone within the tumor. Taken together, our results show the great utility of MSCs in cell/gene therapy, regardless of the pathology. Particularly, in case of cancer, future studies should take our results into account and should test the safety of such therapies for clinical use, especially in patients with other pathologies.

## 4. Materials and Methods

### 4.1. Cell Cultures

Mouse mesenchymal stem cells (mMSCs) were obtained from the femur and tibiae bone marrow (mBMSCs) of 6-week-old female mice Balb/c (Harlan Iberica, Barcelona, Spain), purified by centrifugation (1300 rpm for 15 min at 4 °C) and plated at a seeding density of 1 × 10^6^ cell/cm^2^. Mouse MSCs were grown in MesenCultTM basal media (StemCell Tecnologies Inc., VBC, Canada) containing 10% of Mouse Mesenchymal Stem Cell Stimulatory Supplements (StemCell Tecnologies), 100 unit/mL of penicillin, and 100 g/mL of streptomycin (Invitrogen Life Technologies, Madrid, Spain) at 37 °C, 5% CO_2_ and hypoxic conditions. Three days later, the cultures were washed twice with PBS, the nonadherent cells were removed, and monolayers of adherent cells were cultured until they reached confluence. Cells were then trypsinized (0.25% trypsin with 0.1% EDTA), and subcultured at densities of 5000–6000 cells/cm^2^. Cell cultures at passages 3 and 4 were used for the experiments.

All the other human MSCs (hMSCs) were obtained and cultured as described by Belmar-Lopez et al. [22]. In summary. BM-hMSCs were obtained from Lonza and maintained in DMEM low glucose (1.0 g/L) and hypoxic conditions (3% O^2^). Human epithelial endometrium-derived stem cells or hEESCs (also known as endometrial epithelial stem cell lines; ICEp) were supplied by Dr. Carlos Simon from IVI (Valencia, Spain). Cells were maintained in DMEM F-12 under hypoxic conditions (3% O^2^) and dishes were pre-treated with 0.1% gelatin solution (SigmaAldrich Chemie GmBh, Munich, Germany). Human amniotic membrane mesenchymal stem cells or hAMCs were obtained from Cellular Engineering Technologies (CET), (Coralville, IA, USA) and were maintained in DMEM high glucose (4.5 g/L) and 10 ng/mL basic human fibroblast growth factor (hFGFb; Gibco, Paisley, UK). Cells were used between passages 5 to 8. The hiPSCs (human IPSC line 2 F8) were kindly supplied by Dr. Austin Smith (University of Cambrige, UK) and cultured in knockout DMEM (Gibco., Paisley, UK), 15% knockout serum (Gibco., Paisley, UK), 1× NEAA (Lonza Iberica, Barcelona, Spain), 0.1 mmol/L β-mercaptoethanol (Sigma-Aldrich, Madrid, Spain)10 ng/mL hFGFb, and antibiotics at 37 °C in 5% CO^2^. Cells were seeded on a PN3 feeder cell monolayer inactivated with mitomycin C (Sigma-Aldrich, Madrid, Spain).

A human cervical carcinoma cell line HeLa was obtained from the research cell service of Cancer Research UK Clinical Center (London, UK). A549 cells were obtained from Cell Culture Core, Cancer Research (London, UK). HeLa and A549 cells were grown and maintained at 37 °C with 5% CO_2_ and normoxic conditions in Dulbecco Modified Eagle Medium (DMEM; Invitrogen Life Technologies) containing 10% fetal bovine serum (Lonza, Basel, Switzerland), penicillin (250 U/mL), streptomycin (250 µg/mL), and L-glutamine (2 mM) (Invitrogen Life Technologies).

The cell line TE671-LoxP/LacZ (Allele Biotech, San Diego, CA, USA), of epithelial morphology, was originated from human rhabdomdomoarcoma cultures, generated with a lentiviral system to integrate the loxP/LacZ cassette and cultivated under the same conditions as Hela cells.

### 4.2. Adenoviral and Lentiviral Vectors

Adenovirus Ad-CMV-GFP (Ad-GFP) was obtained from Obiogene (Cambridge, UK), and Ad-CMV-Luc (Ad-fLuc firefly luciferase) from Vector BioLabs (Philadelphia, PA, USA). Adenovirus Ad-CMV-hNIS (Ad-hNIS) was constructed and amplified as previously described [59]. MLV-Lentivirus expressed GFP, also regulated by CMV promoter MLV-LV-CMV-GFP (Lv-GFP), was described previously [60] and was kindly supplied by Dr. Tuan Huy Nguyen (INSERM, Nantes, France). Cre Adenovirus Ad-CMV-Cre (Ad-Cre), under the control of the CMV promoter, was obtained from Vector BioLabs.

The viral titles used throughout the study were between 3.6–4.5 × 10^10^ pfu/mL. After several rounds of amplification, viruses were purified on CsCl gradient [61] and tittered by plaque assay [62].

### 4.3. In Vitro Infection Assay of MSCs by Using Adenoviral Vectors

MSCs were seeded in a 24-well plate at a density of 5 × 10^4^ cells /well. The efficiency at different multiplicities of infection was tested at 48 and 72 h after infection of the cells with each of the adenoviruses (Ad-hNIS, Ad-GFP, Ad-fLuc, and Ad-Cre viruses). To determine this efficiency, each vector was tested in different ways: by a ^125^I uptake assay (Ad-hNIS), by fluorescence microscopy (Ad-GFP), by IVIS scanner (Ad-Luc) and by β-galactosidase staining (Ad-Cre). Each infection was done in triplicate. While Ad-hNIS, Ad-GFP and Ad-Luc tests were performed together using mBMSCs, Ad-Cre in vitro infection assay was done apart, since it was used in BM-hMSCs fusion assays.

### 4.4. Iodide Uptake Assay

Cells were assayed as described [63]. Briefly, after 72 h post-infection, each well was exposed to a PBS solution supplemented with 140 mM NaCl, 20 μM IK, and ^125^I (Specific activity 100 μCi/μmol I) [64] during 1 h at 37 °C in a humidified atmosphere. Control wells were exposed to an additional 100 μM NaClO_4_ to inhibit hNIS-mediated iodine accumulation. I- accumulation finished after removing uptake solution and washing twice with 1 mL of ice-cold PBS. The amount of I- accumulated was determined by adding 500 μL of cold ethanol during 20 min at 4 °C and radio-activity was quantified in Ci a gamma scintillation counter (γ-counter). To normalize results by number of cells, DNA present in each well was determined by diphenylamine method after 5% trichloroacetic acid precipitation.

### 4.5. Infection of MSCs by Using Adenoviral or Lentiviral Vectors

Ad-hNIS, Ad-GFP, Ad-fLuc, Lv-GFP, and Ad-Cre viruses were diluted in culture media with 5% FBS and added to cells using 500 MOI and incubated for 1 h at 37 °C. Then 10% FBS medium was added, and cells were maintained for 24 h until used in in vivo experiments.

### 4.6. In Vivo Experimental Design

#### 4.6.1. Induction of Pathologies

Adult females (of BALB/c nu/nu mice six to eight-weeks old) were obtained from Harlan Iberica. The animals were kept in ventilated cages with free access to standard pellet animal laboratory food and water. Random division of the animals into four groups took place for the parallel study of mBMSCs targeting in control, tumor, diabetes, and wound healing mouse models.

- Diabetes: Mice were fasted for 20 h before diabetes was induced with streptozocin (STZ; Sigma Aldrich, Madrid, Spain). Later, mice received intraperitoneal (i.p.) injection of 150 mg/kg STZ dissolved in 0.1 mol/L citrate buffer (pH 4.5) as previously described [65]. Changes in serum glucose, total cholesterol, and insulin levels after STZ administration were at different time points. Diabetes was assessed by measuring blood glucose levels using an analyzer Glucocard Gmeter, (A. Menarini Diagnostics España, Badalona, Spain).
- Wound healing: Animals undergoing skin injury were anesthetized with i.p. pentobarbital (Sigma Aldrich) and a biopsy, with a punch biopsy was performed. The injury was performed on the previous day to the MSCs transplantation.
- Tumor model: Tumor induction was done with subcutaneous injection of 1 × 10^6^ HeLa cells or TE671-LoxP/LacZ cells resuspended in 200 mL of PBS. These two different tumor xenograft mouse models were induced for different studies: while Hela Tumor xenografts were used for MSCs tracking experiments, ^131^I therapeutic assays and cell fusion experiments, TE671-LoxP/LacZ xenografts were generated to test transdifferentiation mechanisms.

MSCs transplantation was done when tumor size reached 0.5 cm^2^ volume. Growth of tumor was monitored twice a week, and tumor volumes were calculated from caliper measurements of two orthogonal diameters (x and y) using the formula: volume = (1/2)*xy^2^.

#### 4.6.2. MSCs Migratory Ability in Different Pathologies

Once the pathology had been induced, mice coming from the different pathologies were divided into different subgroups. mBMSCs, that had been viral infected 8 h before, were intravenous (i.v.) injected at a dose of 106 resuspended in 200 mL of PBS via the lateral tail vein:

1. Eight days after diabetes induction, mice received i.v. injection of MSCs-Ad-CMV-fLuc and i.v. injection of mBMSCs-LV-CMV-GFP.
2. Injury/wound healing mice received i.v. injection of mBMSCs-Ad-CMV-hNIS or i.v. injection of mBMSCs-LV-CMV-GFP.
3. Ten days after HeLa tumor cell injection, when mice have developed macroscopic tumor nodules, mice received i.v. injection of mBMSCs-Ad-CMV-hNIS or i.v. injection of mBMSCs-LV-CMV-GFP.
4. Control group animals received i.v. injection of 200 µL of PBS (without MSCs).

Forty-eight hours later, some animals received a 1.85 Mbq of Na^124^I injection intravenously to be PET scanned. Mice treated with mBMSCs-Ad-hNIS were scanned repeatedly in SPECT/CT scanner, while mice injected with mBMSCs-Ad-fLuc were scanned in the IVIS. Mice injected with mBMSCs-Lv-GFP were sacrificed, and their tumors and organs were analyzed by PCR, Western blot, and confocal microscopy.

#### 4.6.3. In Vivo Therapeutic Effect of ^131^I

Animals coming from the Hela tumor induced mice were divided. First group animals received i.v. injection of 10^6^ mBMSCs infected with Ad-hNIS 8 h before (mBMSCs-hNIS group). Group 2 received the same i.v. injection of 10^6^ mBMSCs-hNIS cells and subsequent intraperitoneal injection of a 2 mCi dose of ^131^I (mBMSCs-hNIS + ^131^I group). The third group received i.v. injection of 200 µL of PBS. Finally, group 4 received i.v. injection of 200 µL of PBS and a subsequent i.p. injection at a dose of 2 mCi of 131I (PBS group + ^131^I) (see Figure 5A).

Animals followed a low iodide diet and L-thyroxine (T4) [66] at a dose of 5 mg/L in water 7 days before radioisotope administration to prevent thyroid damage, and endogenous hNIS signal Treatment was also initiated when Hela tumor reached a volume of 0.5 cm^2^. The monitoring of the growth of the tumor in each group was done as described above.

#### 4.6.4. In Vivo MSCs Asserting Mechanisms

The main difference of these studies was the use of BMhMSC in cell fusion experiments and the administration of human mesenchymal stem cells from diverse lineages in transdifferentiation assays. On the one hand, once subcutaneous cell tumors TE671-LoxP/LacZ reached the desired size, animals were randomly separated into three groups: trial group BMhMSCs-Cre, Ad-CMV-Cre group, and control group. Trial group mice received i.v. injections into the lateral vein of the tail of 106 BMhMSCs previously infected with an Ad-Cre. The control group received an intravenous injection of 200 µL of PBS. Ad-Cre animals received intratumoral injection of 10^8^ pfu of Ad-Cre resuspended in 100 µL of PBS. Then, tumors were analyzed by histology and RT-PCR to explore BMhMSCs-Cre- TE671-LoxP/LacZ fusion.

On the other hand, a group of mice coming from Hela tumor model was separated into four test groups: hEESCs-hNIS group, hASCs-hNIS group, hiPSCs-hNIS group, and a control group. The animals in each test group received i.v. injections into the lateral vein of the tail of 10^6^ cells from each of the lines of hMSCs previously infected with an Ad-hNIS at 500 MOIs. The animals in the control group received an intravenous injection of 200 µL of PBS. Then, tumors were processed sectioned and stained with hematoxylin-eosin to explore hMSCs transdifferentiation.

In each study group, random mice were sacrificed by cervical dislocation at different times. Tumors and other organs were excised and weighed. Then tissues, collected for different studies, were frozen and stored at −80 °C.

### 4.7. Imaging Techniques

#### 4.7.1. Bioluminescence Imaging

Optical imaging was performed using the Xenogen In vivo Imaging System (IVIS; Caliper Life Sciences, Hopkinton, MA, USA). The system consists of a supersensitive cooled charge-coupled device (CCD) camera mounted inside a light-tight imaging chamber to capture both a visible light photograph of the animal taken with light-emitting diodes and the luminescent image.

Images were acquired at 1- to 10-min intervals until the peak signal was observed. The grayscale photographic images and bioluminescence color images were superimposed using the Living Image V 2.6.1 software overlay (Caliper Life Sciences, Waltham, MA, USA). For quantification, the region of interest (ROI) was manually selected based on the signal intensity emitted. The area of ROI was kept constant, and the intensity was recorded as average photons per second per square centimeter per steradian (p/s/cm^2^/sr) [67].

To visualize the adenovirus Ad-fluc in the infection assay, 100 μL from D-Luciferin (Caliper Life Science, Hopkinton, MA, USA) final concentration 150 μg/mL were added to each well. To monitor the distribution of mBMSCs-CMV-Luc, we injected D-luciferin firefly potassium salt substrate at a dose of 150 mg/kg body weight in 300 µL PBS intraperitoneally. General anesthesia was then induced with 5% isoflurane, and the mouse was placed in the light-tight heated chamber with 2% isoflurane introduced via cone. The animals were imaged over a 20-min time period with 1-min acquisition intervals using the IVIS system.

#### 4.7.2. Nano-SPECT/CT Imaging

After anesthesia (i.p. injection of ketamine/xyzaline; 1.5 mL/kg, ratio 2:1), mice were injected with 18.5 MBq of ^99m^TcO_4_^−^ and scanned in a SPECT/CT scanner (Bioscan, Washington DC, USA). SPECT and CT images were acquired with different timing based on specific radioactive levels to obtain 100,000 c.p.s. Images were reconstructed with MEDISO software (Medical Imaging Systems, Budapest, Hungary) and fusion using PMOD software (PMOD Technologies Basel, Switzerland).

### 4.8. Histology

#### 4.8.1. Hematoxylin-Eosin Staining and Immunohistochemistry

Histology was performed on 4 mm sections collected at different time points, formalin-fixed, paraffin-embedded tissues for hematoxylin-eosin staining, and β-galactosidase staining. Images were acquired using an Olympus BX51 microscope (Olympus) and Studio Lite software (Li-COR Biosciences, GmbH).

The hematoxylin-eosin staining was performed by means of a Leica ST5020 (Barcelona, Spain) and CV5030 automatic dye and mounter. To this end, the preparations posed as successive solutions of xylene, alcohols in decreasing gradation (100, 96, and 70%) to hydration, Carazzi hematoxylin, running water, eosin and 1%, increasing alcohol solutions up to complete dehydration (70, 96, and 100%) to finish with the rinsing in two xylene baths and assembly with permanent medium (Sigma-Aldrich).

Immunohistochemistry was performed on 4-μm sections of formalin-fixed, paraffin-embedded tissues. The antibody used was anti-human NIS (hNIS) (rabbit polyclonal) (1:2000), kindly donated by Dr. de la Vieja.

#### 4.8.2. Detection of LacZ by β-Galactosidase Staining

Sigma-Aldrich’s GALS commercial kit was used in the stained-of-galactosidase (LacZ gene of the operon lac). For cell staining directly on the plate, the cells were fixed with 1X fixing solution (2% formaldehyde, 0.2% glutaraldehyde in PBS 1X) for 10 min at room temperature and washed with PBS. Subsequently, they were incubated with the X-Gal staining solution (2 mM MgCl_2_, 4 mM potassium ferricyanide, 4 mM potassium ferrocyanide and 1 mg/mL X-Gal in dimethylformamide (DMF), in PBS 1X) at 37 °C for 2 h or longer until blue coloring was observed. For tissue staining, the samples were washed in PBS at 4 °C and immediately fixed at 4% paraformaldehyde in PBS for 3 h at 4 °C. Then three washes of 30 min each were performed with washing solution (100 mM sodium phosphate at pH 7.3, 2 mM MgCl_2_ to, 0.01% sodium deoxycholate and 0.02% NP-40) to the X-Gal staining solution was added to samples (5 mM potassium ferricyanide, 5 mM potassium ferrocyanide and 1 mg/mL X-Gal in washing solution), incubating them for 16 h. Finally, they were set in 4% paraformaldehyde (PFA) at 4 °C for 16 h and incubated in 70% ethanol before being processed.

### 4.9. DNA Extraction and PCR Analysis

Genomic DNA was extracted using DNA isolation kit (NucleoSpin Tissue, Macherey-Nagel GmbH &Co., Düren, Germany). PCR reaction was performed using a Master Mix (Promega, WI, USA) following the manufacturer instructions and analysis using GFP-specific primers, forward 5′- TGAGCAAGGGCGAGGAGC -3′ and reverse 5′-GGAATTCCATATTTGTACAGCTCGTCCATGCCG-3′, GAPDH-specific primers, forward 5′-TGAAGGTCGGTGTGAACGGATTTGGC-3′ and reverse 5′-CATGTAGGCCATGAGGTCCACCAC-3′ (Sigma-Aldrich). Cycling was done at 95° C for 5 min, followed by 35 cycles of 95 °C for 1 min, 60 °C for 1 min and 72 °C for 1 min, and a final elongation reaction of 72 °C for 10 min. DNA products were separated in 1% agarose gel (Lonza, Rockland, ME, USA), stained with SYBER (ThermoFisher Scientific, Pittsburg, CA, USA) and visualized and pictures under UV illumination using G-Box Syngene system (Synoptics Group, Frederick, MD, USA).

### 4.10. RNA Extraction and RT-PCR

The NucleoSpin^®^RNA II commercial kit (Macherey-Nagel) was used for the extraction of total RNA from 10–15 mg of tissue following its instructions. To ensure that there was no contamination of genomic DNA and starting with 2 μg of RNA, a solution of 2 U (1 U/μL) of DNase I (Fermentas Inc., Hanover, NH, USA) was added and incubated for 1 h at 37 °C. Once done, in order to inactivate DNase I, 2 μL of 25 mM EDTA was added to the solution and incubated for 10 min at 65 °C. Reverse transcriptase SuperScript II (Invitrogen Life Technologies) was used to retrotranscribe the RNA to cDNA. The following procedure was: (i) addition of 3 μg of random primers (Invitrogen Life Technologies) and 1 μL of 10 mM dNTP’s (Invitrogen Life Technologies) to 10 μL of the previous RNA/DNase solution. This mixture was incubated for 5 min at 56 °C; (ii) addition of 5 µL of a ribonuclease inhibitor solution (40 U) or RNasaOUT (Invitrogen Life Technologies) to the above solution that incubated for 2 min at room temperature; (iii) addition of 3 µL of a reverse transcriptase solution (200 U) and incubation for 10 min at room temperature. The reaction control was done in parallel without the reverse transcriptase added (called FRT; false retrotranscription). Finally, the mixture was incubated 1 h at 42 °C, and it was followed for other incubation of 15 min at 70 °C. The resulting cDNA solution (50 ng/μL) was kept at −20 °C until use.

### 4.11. Protein Extraction and Western Blot Analysis

Mice tissues were homogenized on 100 µL of lysis buffer containing 150 mm NaCl, 50 mM Tris pH 7.5, 0.05% sodium dodecyl sulfate, 1% Triton X-100, sonicated on ice and centrifuged to obtain whole protein extract. Western blot analysis was performed as previously described [66]. Primary antibodies anti-GFP (N-ter; Cancer Research-UK antibodies service) or anti-beta-tubulin (Sigma Aldrich) were used according to the manufacturer’s instructions. Pierce ECL Western blotting substrate was used to visualize the immunoreactive bands (ThermoFisher Scientific).

### 4.12. Fluorescence and Confocal Microscopy

Sections of 6–8 µm were obtained from tissues and frozen tumors. Infected mBMSCs, seeded at the density mentioned above, were directly visualized after 48 h. Imaging was performed with confocal/microscopy equipped with a CCD camera and analysis software. Image acquisition by confocal microscopy was carried out using the Leica TCS SP2 AOBS confocal microscope. The images were taken using the microscope in sequential mode, 40×, and/or 63× oil immersion objectives (lens specification: HCXPLAPO NA 1.25 and HCXPLAPO NA 1.40, respectively), a number of 16 passes per line and a 1024 × 1024-pixel format. The confocal or pinhole opening was set on an Airy unit. The images were analyzed with the LCS 1537, software version 15.37 (Leica).

### 4.13. Statistical Analysis

Numerical data were expressed as means, including standard error. Statistical differences between means for different conditions were evaluated with Prism 4.0 (GraphPad Software, CA, USA) using either the Student’s *t*-test or analysis of variance (ANOVA), with a significance *p* < 0.05.

### 4.14. Ethical Approval

All experiments were conducted with appropriate ethical approval of the Ethics Committee for Animal Experiments from University of Zaragoza (Spain) and accordance with the Guidance on the Operation of the Animal (Scientific Procedures) Act 1986 (House of common 1990). License ref PI15/08 Care and use of animals were carried out according to the Spanish Policy for Animal Protection RD1201/05.

## 5. Conclusions

This study confirms the migration potency of MSCs to damaged tissues in three different pathologies. Clinically, this work provides evidence for future development of real-time in vivo stem cell-based therapies in different diseases, especially in individuals with more than one inflammatory pathology. The application of the MSCs therapies in individuals with several inflammatory pathologies at the same time should be taken in special consideration and it should be studied in detail.

Moreover, this work highlights hNIS as a useful therapeutic tool when radioisotope ^131^I is administrated, either alone or combined with adenoviral vectors. Finally, this research provides further understanding of MSCs homing mechanisms. Although these mechanisms are not yet fully understood, here we discarded fusion events and confirmed the ability of MSCs to change into osteoblasts, at least in our model. In summary, the parallel discovery of MSCs migration in different mouse model diseases, their different arrival times depending on the pathology, and their unclear mechanism of action reinforce them as particularly interesting candidates for further investigation.

## Figures and Tables

**Table 1:** Subgroups of animals according to the engineered mBMSCs administration and the pathology induced.

	Diabetes Model	Injury/Wound Healing Model	Hela Tumor Model	Control Group
A	mBMSCs-Ad-CMV-fLuc	mBMSCs-Ad-CMV-hNIS	mBMSCs-Ad-CMV-hNIS	PBS
B	mBMSCs-Lv-CMV-GFP	mBMSCs-Lv-CMV-GFP	mBMSCs-Lv-CMV-GFP	

**Table 2:** Subgroups of Hela tumor induced animals according mBMSCs-hNIS/ mBMSCs-hNIS + 131I administration.

	Group 1	Group 2	Group 3	Group 4
MSCs infected with	mBMSCs-Ad-CMV-hNIS	mBMSCs-Ad-CMV-hNIS	PBS	PBS
^131^I administration	NO	2 mCi ^131^I	NO	

**Table 3:** Average of tumor sizes (mm3) at day 15 and 37 in the different groups of mice administered with mBMSCs-hNIS or PBS and treated with 131I or PBS.

Day	Group 1 (PBS)	Group 2 (PBS + ^131^I)	Group 3 (mBMSCs-hNIS)	Group 4 (mBMSCs-hNIS + ^131^I)
15	122.20 ± 5.86	95.21 ± 12.97	82.40 ± 10.22	60.15 ± 17.12
37	182.78 ± 4.99	154.01 ± 12.27	173.53 ± 11.87	60.16 ± 10.08

**Table 4:** Subgroups of TE671-LoxP/LacZ tumor induced animals according to transgenic Cre administration.

	Group 1	Group 2	Group 3
Type of Transgenic Cre administration	BMhMSCs-Cre	Ad-CMV-Cre	PBS

## Data Availability

Data sharing is not applicable to this article.
